# Added value of patient-reported outcome measures (PROMs) after an acute stroke and early predictors of 90 days PROMs

**DOI:** 10.1186/s41687-022-00472-9

**Published:** 2022-06-13

**Authors:** Ester Sanchez-Gavilan, Estefania Montiel, Maria Baladas, Sofia Lallanas, Eva Aurin, Carolina Watson, Maria Gutierrez, Yolima Cossio, Marc Ribo, Carlos A. Molina, Marta Rubiera

**Affiliations:** 1 Stroke Unit - HARMONICS research group, Hospital Universitari Vall d’Hebron, Vall d’Hebron Barcelona Hospital Campus, Passeig de la Vall d’Hebron, 119-129, 08035 Barcelona, Spain; 2 Value Based Health Care, Hospital Universitari Vall d’Hebron, Barcelona, Spain; 3 Innovation and Digital Health, Hospital Universitari Vall d’Hebron, Barcelona, Spain

**Keywords:** Depression, Anxiety, Stroke, PROMs, Outcomes, Value based health care, Health care system

## Abstract

**Introduction:**

Value-based health care represents a patient-centered approach by valuing Patient-Reported Outcome Measures (PROMs). Our aim was to describe the additional value of PROMs after an acute stroke over conventional outcome measures and to identify early predictors of poor PROMs.

**Methods:**

Acute stroke patients discharged from a tertiary care hospital followed by a web/phone-based PROMs collection program in the post hospitalization phase. Main PROMs involve anxiety and depression (HADS) (each defined by HADS ≥ 10) and global physical (PHY-) and mental (M-) health (PROMIS-10). PROMIS cut-off raw values of normality were: PHY-PROMIS ≥ 13 and M-PROMIS ≥ 11. An overall health status (OHS) from 0 to 100 was also determined. PROMs related to the different modified Rankin Scale (mRS) grades were defined. Early predictors of PROMs were evaluated.

**Results:**

We included 1321 stroke patients, mean age 75 (± 8.6) and 55.7% male; 77.7% returned home. Despite a favorable mRS at 3 months (< 3), a relevant rate of patients considered without symptoms or with mild disability showed unfavorable results in the measured PROMs (8% unfavorable OHS, 15% HAD-depression, 12.1% HAD-anxiety, 28.7% unfavorable M-PROMIS and 33.1% unfavorable PHY-PROMIS results). Along follow-up, only PHY-PROMIS and OHS showed significant improvement (p < 0.01 and 0.03, respectively). The multivariate analysis including discharge variables showed that female sex, higher discharge mRS and discharge to socio-rehabilitation-center (SRC) were independent predictors of unfavorable results in PROMs (p < 0.01). When adding 7 days PROMs results, they emerged as the strongest predictors of 3 months PROMs.

**Conclusions:**

A high proportion of stroke patients show unfavorable results in PROMs at 3 months, even those with favorable mRS, and most results obtained by PROMs during follow-up continued to indicate alterations. Female sex, mRS and discharge to SRC predicted unfavorable results in PROMs, but the strongest predictors of 3 months PROMs were the results of the 7 days PROMs.

## Introduction

Stroke is one of the most devastating diseases of the world. There are over 12.2 million new strokes each year, and over 101 million people currently living who have experienced stroke. Globally, one in four people over age 25 will have a stroke in their lifetime [1]. It represents the leading cause of long-term adult disability and the second leading cause of death by specific entities worldwide [2]. Furthermore, among stroke survivors, a high proportion will present permanent impairments resulting in deficient self-care and the need to be supported on the long term by caregivers leading to massive individual, healthcare and social costs. Over 143 million years of healthy life is lost each year due to stroke-related death and disability [1].

For years, evaluation of healthcare organizations has been based on the number of delivered services [3], but this is changing over time. In the early 1960s, psychologist Carl Rogers was the first to use the term ‘person-centred’, in relation to psychotherapy (and had used ‘client-centred’ as early as the 1950s) [4]. Years later, in 2010, M. Porter established the base of a new era in health care by putting patients and what they think is valuable in the center of medicine. Since then, value-based health care (VBHC) is becoming the new paradigm of clinical management [5]. Knowing what is valuable for the patient and ensuring good coordination between clinical professionals and the healthcare network is the key for better care and controlled costs [6, 7].

VBHC copes with the aging population and the increase in chronic diseases including stroke. This new vision places the patients and their state of health (disease) at the center of the intervention, considering what is relevant for them by determining Patient-Reported Outcome Measures (PROMs). VBHC aims to achieve the best health results at the lowest possible cost [7, 8].

One of the main challenges of VBHC is the selection and recollection of PROMs. The International Consortium of Health Outcome Measurement (ICHOM) is a non-profit organization of experts who defined standard sets of PROMs that matter most to patients for different health conditions, such as stroke [9]. Thanks to these Standard Sets, value can be measured using PROMs worldwide for all stakeholders, and benchmarking can be performed. On the other hand, collection of PROMs requires an inter-connection between patients and healthcare providers that may be favored by the Information and Communication Technologies (ICTs), in order to offer the best communication tool possible.

Our aim was to establish a PROMs collection program in stroke patients discharged from a Comprehensive Stroke Center. We also aimed to demonstrate the added value of PROMs over the most usual outcome measure in acute stroke, the modified Rankin scale. Finally, we tried to identify predictive factors during hospitalization and early phase after discharge associated with poor PROMs at 90 days.

## Methods

This is a prospective study of consecutive acute stroke patients discharged from the stroke Unit at the Hospital Universitari Vall d’Hebron following a web/phone-based PROMs collection program.

The VBHC stroke program (Value Based Stroke Care, VBSC) of the Hospital Universitari Vall d’Hebron was designed in 2018. After several meetings with patients, relatives and multidisciplinary health care workers involved in stroke care, a set of PROMs were selected in concordance with the ICHOM Standard Sets for stroke. Data collection and management was planned to be carried out by a specifically designed web-based platform.

From August 2018 till May 2020, all consecutive stroke patients admitted to our Stroke Unit and later-on discharged to home or to a socio-rehabilitation center (SRC) were included. The only exclusion criteria were language barrier, patients resident in another country who would return to their country of origin, or patients discharged to another acute care hospital.

Before discharge, patients and/or their relatives were contacted by a Stroke Process Manager (SPM), who explained the VBSC and obtained the signed informed consent to participate in the program. After inclusion, patients were entered into an online platform and they received follow-up during one year. A personal e-mail address and/or telephone contact was obtained from patients/next of kin and used to collect further PROMs. E-mails including links to the standardized PROMs scales were sent according to a predefined follow-up schedule. The language used to deliver the scales was Spanish. When e-mail contact was not available, a telephone interview with the SPM was carried out to fill the same scales following the same temporal schedule. Additionally, an out-patient visit was scheduled after 3–4 months from stroke to evaluate clinician-evaluated functional outcome by the modified Rankin scale.

The set of scales to obtain PROMs and their timing are shown below:

- Patient-Reported Outcomes Measurement Information System 10 (PROMIS-10) [10]: ten items scale that assess physical function, pain, fatigue, emotional distress, social health, and general perceptions of health. Results are encompassed in 2 sub-scores, Global Physical Health Score (PHY-PROMIS) and a Global Mental Health score (M PROMIS). Results are considered as absolute numbers (range between 4 and 20, higher scores, more favorable outcomes). They can also be relative to a T value conversion process established according to the results of a general (normal) American population. Scores in the T-value table lower than one-standard deviation below 10 points the T50 are considered pathological. According to this T conversion, punctuations below 13 on PHY-PROMIS and below 11 on M-PROMIS are considered as poor outcomes [8]. PROMIS-10 was obtained after 7 days from discharge and 3 and 12 months after stroke.
- Hospital Anxiety and Depression Scale **(**HADS) [11]: 14-item scale; 7 items provide information about anxiety (HADS-anxiety) and 7 about depression (HADS depression). Results are depicted as absolute numbers (range between 0 and 21; higher scores, unfavorable outcomes). Score equal or higher than 10 in both sub-scales indicate anxiety and depression determined by HADS. HADS was obtained after 3 and 12 months from stroke.
- Overall health status (OHS): numerical scale from 0 to 100 to auto-evaluate the global health self-perception (higher score, more favorable outcome). Evaluated after 7 days, 3 and 12 months.

In addition to PROMs, baseline clinical characteristics, stroke subtype, discharge destination and clinical and functional outcomes (baseline and discharge National Institute of Health Stroke Scale (NIHSS) and modified Rankin scale (mRS)) at discharge and after 3 months were also recorded by the medical team. NIHSS is the most used scale globally nowadays to evaluate basic neurological functions in the acute phase of stroke, but it does not accurately detect common symptoms of posterior circulation strokes [12].

### Statistical analysis

Categorical variables are presented as absolute values and percentages, and continuous variables as mean ± standard deviation (SD) if normally distributed or median and interquartile range (IQR) if not normally distributed. Statistical significance for intergroup differences was assessed by Fisher’s exact test for categorical variables and by Student *t* or Mann–Whitney *U* test for continuous variables. The Wilcoxon Signed Rank test was used to evaluate correlation between non-normally distributed continuous variables. Two multivariable logistic regression models were performed for each PROM to determine factors that could be considered as independent predictors of poor results in PROMs at 3 months. For the OHS, given that it is a continuous variable with normal distribution, we considered poor OHS values lower than 1 SD below the mean OHS (<40). The first model included discharge clinically relevant variables. The second model added the results of PROMs acquired at 7 days after discharge in the analysis. Variables showing p<0.1 in univariate analysis were included in the multivariate model. A two-sided p<0.05 was considered significant for all tests. All statistical analyses were carried out using IBM SPSS 25.0 software (IBM Corporation, Armonk).

## Results

From August 2018 to May 2020, 1670 patients were admitted to our Stroke Unit. After excluding patients with final diagnosis of stroke mimic (n = 2), patients transferred to other acute hospitals (176), those with language barrier or foreign residents who would return to their country of origin (4) and patients who died during hospitalization (133), 1355 patients were offered to participate in our PROMs recollection program. Thirty-four of them did not consent, and therefore, 1321 (97.5%) patients were included in the study.

The baseline clinical characteristics of the patients are shown in Table 1. One-thousand and sixty-four patients (80.5%) were ischemic strokes, 106 (8%) were acute intracerebral hemorrhage and 146 (11.1%) were transient ischemic attacks (TIA). Five (0.4%) patients presented with an acute cerebral venous thrombosis. The median time of hospitalization was 5.6 days (3.2–12.7), and of all patients, 3 out of 4 were discharged home versus discharged to SRC.

**Table 1:** Baseline clinical characteristics and main PROMs results

Demographics (n = 1321)	
Age, mean(SD)	75 (8.6)
Sex (male), n (%)	736 (55.7)
Baseline mRS, median(IQR)	1 (0–2)
Baseline NIHSS	5 (1–10)
Discharge NIHSS	1 (0–4)
Discharge mRS	2 (1–3)
*Discharge destiny*	
Home	1027 (77.7%)
SRC	294 (22.3%)
3 months mRS	2 (1–3)
Independent at 3 months (mRS < 3)	452 (35.1%)

PROMs	7 days (n = 900)	90 days (n = 671)	1 year (n = 177)
PHY-PROMIS, median (IQR)	12 (10–14)	13 (10–16)	13(11–16)
Poor PHY-PROMIS (< 13)	504 (56%)	327(48.7%)	73 (41.2%)
M-PROMIS, median (IQR)	12 (9–14)	12 (9–14)	12 (9–14)
Poor M-PROMIS (< 11)	339 (37.7%)	264 (39.3%)	59 (33.3%)
HADS-depression, median (IQR)		6 (2–12)	6 (2–10)
HADS-depression ≥ 10		205 (30.8%)	47 (26.6%)
HADS-anxiety, median (IQR)		6 (3–9)	6 (3–8)
HADS-anxiety ≥ 10		143 (21.5%)	35 (19.8%)
OHS, mean (SD)	59.9 (24.1)	62.9 (23.3)	66.1 (20.3)

*PROMs* patient reported outcomes; *NIHSS* National Institute of Health Stroke Scale (0 no stroke symptoms-42 severe stroke); *mRS* modified Rankin Scale (0 no disability-6 death); *SRC* socio-rehabilitation center; *IQR* interquartile range; *SD* standard deviation; *PHY-PROMIS* physical sub-score of Patient-Reported Outcomes Measurement Information System 10 (PROMIS-10) (range: 4 (worst perception of health status)–20 (best perception of health status), considered altered < 13); *M-PROMIS* metal sub-score of PROMIS-10 (range: 4 (worst perception of health status)–20 (best perception of health status), considered altered < 11); *HADS* Hospital Anxiety and Depression Scale (range: 0 (normal)–21 (worse score), considered altered ≥ 10); *OHS* overall health status (range: 0 (worst perception of health status)–100 (best perception of health status))

**Fig. 1:**
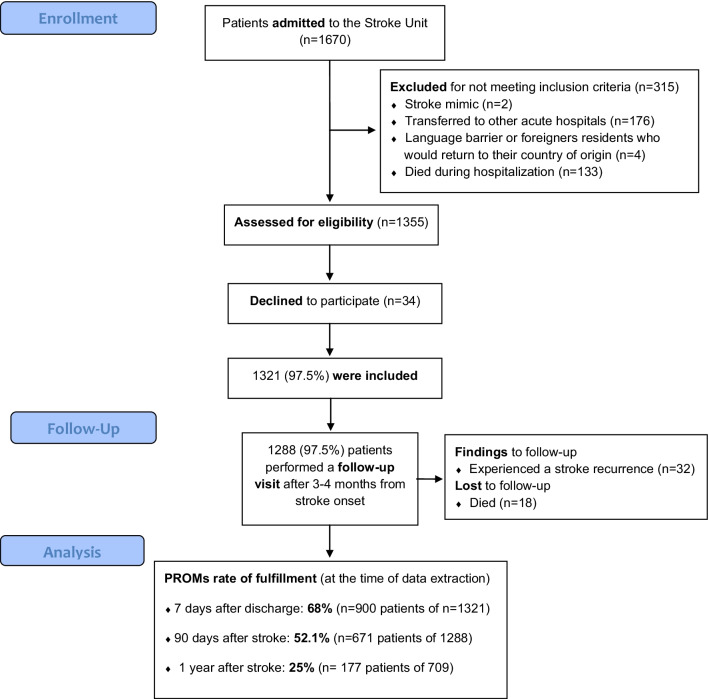
Flowchart of all patients admitted to the Stroke Unit and PROMs fulfillment rate at the time of data extraction of patients that were finally included

An out-patient follow-up visit was performed after 3–4 months from stroke onset in 1288 patients (97.5%). The median mRS evaluated at 3 months follow-up was 2 (1–3). Four hundred and fifty-two (35.1%) patients were functionally independent at 3 months (mRS<3), 32 (2.5%) patients experienced a stroke recurrence and 18 (1.4%) patients died during follow-up. PROMs survey completion rate decreased with time from discharge: after 7 days, 900 of 1321 (68%) patients completed the tests; at 3 months, 671 of 1288 (52.1%) and after one year, only 177 of 709 (25%) patients who reached the one year follow-up time-point and received the questionnaires answered them (Fig. 1).

Table 1 also shows the global results of PROMs. At 3 months, 48.7% patients reported unfavorable results in PHY-PROMIS, 39.3% showed affected mental outcomes in M-PROMIS, 30.8% presented with HADS-detected depression and 21.5% with anxiety in HADS sub-scale. The mean OHS was 62.9 (±23.3), 0 being the worst perception of quality of life and 100 being the best perception. Two-hundred eighty-eight (43%) patients showed favorable results in all evaluated PROMs; 17.1% presented with 1 unfavorable outcome, and the remaining 39% of patients reported unfavorable results in 2 or more PROMs. Thirty-three (5%) patients reported an unfavorable outcome in all measured PROMs.

**Fig. 2:**
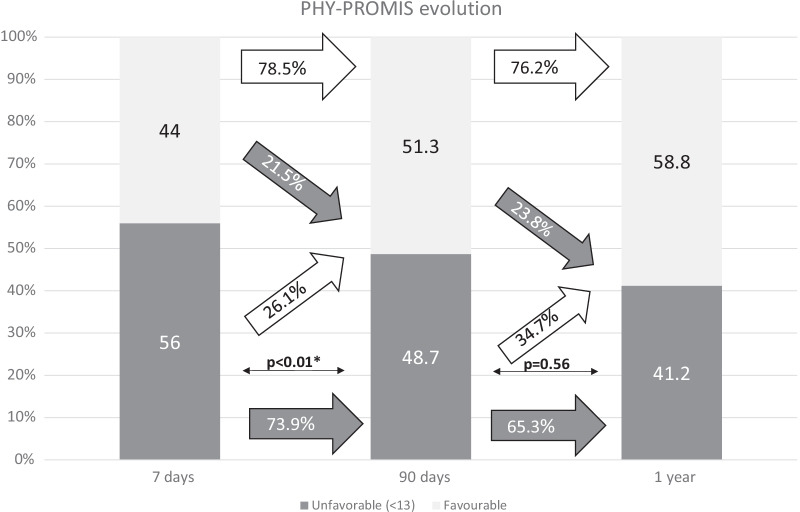
Rate of favorable and unfavorable results in the physical sub-score of PROMIS-10 scale (PHY-PROMIS) at different time-points along follow-up: 7 days, 90 days and one year after stroke. The white arrows represent the rate of patients with improvement in the measured PROM or persistence of favorable outcome at the following time-point of evaluation. The gray arrows represent impairment toward an unfavorable result in the measured PROM or unfavorable outcome persistence. Thin arrows represent statistical comparison of the rate of favorable/unfavorable outcomes as compared with the previous time-point. *Statistically significant

**Fig. 3:**
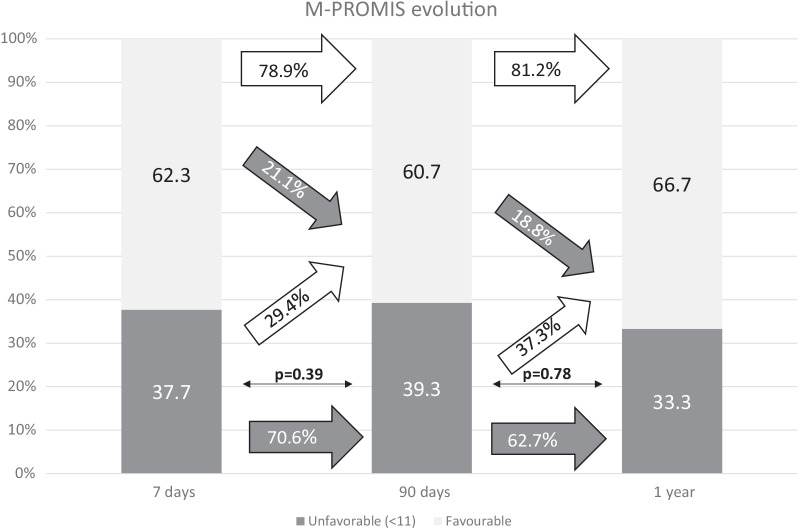
Rate of favorable and unfavorable results in the mental sub-score of PROMIS-10 scale (M-PROMIS) at different time-points along follow-up: 7 days, 90 days and one year after stroke. The white arrows represent the rate of patients with improvement in the measured PROM or persistence of favorable outcome at the following time-point of evaluation. The gray arrows represent impairment toward an unfavorable result in the measured PROM or unfavorable outcome persistence. Thin arrows represent statistical comparison of the rate of favorable/unfavorable outcomes as compared with the previous time-point. *Statistically significant

**Fig. 4:**
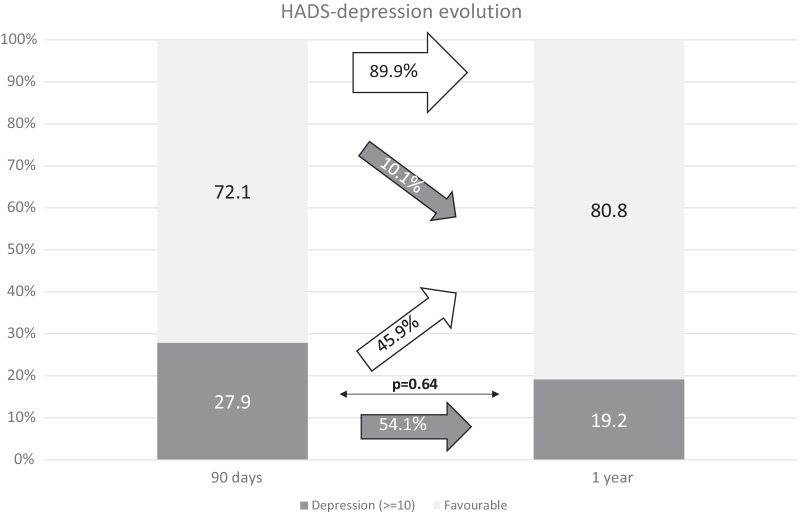
Rate of favorable results in the depression items of HADS scale and depression diagnosed by HADS at different time-points along follow-up: 90 days and one year after stroke. The white arrows represent the rate of patients with improvement in the measured PROM or persistence of favorable outcome at the following time-point of evaluation. The gray arrows represent impairment toward an unfavorable result in the measured PROM or unfavorable outcome persistence. Thin arrows represent statistical comparison of the rate of favorable/unfavorable outcomes as compared with the previous time-point

**Fig. 5:**
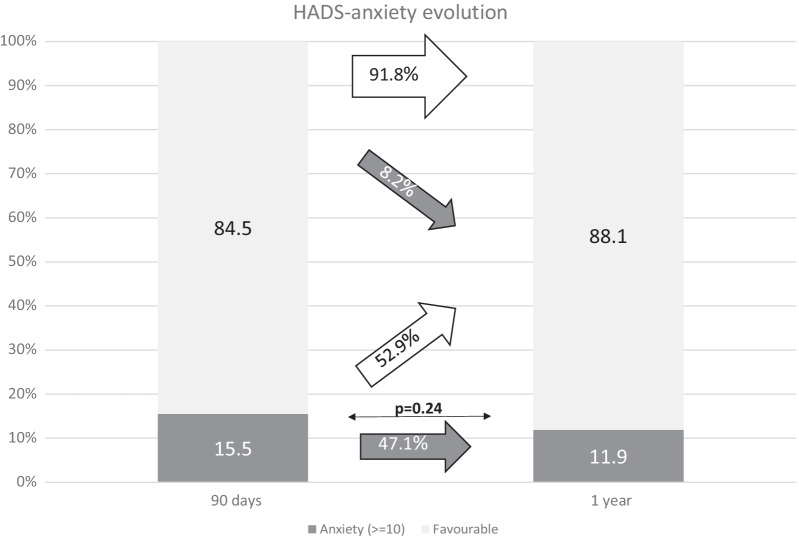
Rate of favorable results in the anxiety items of HADS scale and anxiety diagnosed by HADS at different time-points along follow-up: 90 days and one year after stroke. The white arrows represent the rate of patients with improvement in the measured PROM or persistence of favorable outcome at the following time-point of evaluation. The gray arrows represent impairment toward an unfavorable result in the measured PROM or unfavorable outcome persistence. Thin arrows represent statistical comparison of the rate of favorable/unfavorable outcomes as compared with the previous time-point

**Fig. 6:**
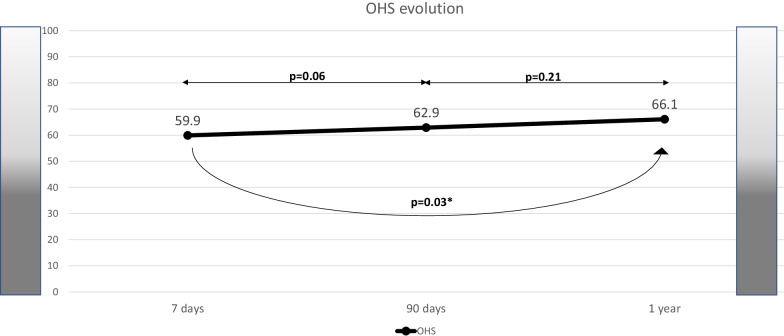
Mean of overall health status (OHS) at the different time-points of measurement (7 days, 90 days and one year after stroke). Range: 0 (worst perception of health status)–100 (best perception of health status). Thin arrows represent statistical comparison of the rate of favorable/unfavorable outcomes as compared with the previous time-point. *Statistically significant

Figures 2, 3, 4, 5 and 6 show the evolution of PROMs favorable and unfavorable results along follow-up. Poor outcomes rates did not decrease notably during follow-up. Only PHY-PROMIS showed a significant statistical improvement from day 7 to 3 months (p < 0.01) and OHS from day 7 to 1 year (p = 0.03); the remaining PROMs did not statistically improve along the follow-up. From all patients reporting depression on HADS at 3 months, 54.1% continued to report depression after 1 year. Patients who reported anxiety at 3 months continued to report it after 1 year in 47.1% of cases. According to PHY-PROMIS, from those reporting unfavorable results in PHY-PROMIS at 3 months, 65.3% still considered having physical deficits detected by PHY-PROMIS after 1 year from stroke onset. The rate of persistence of poor outcomes on M-PROMIS in those reporting it after 3 months was 66.7% after one year.

**Fig. 7:**
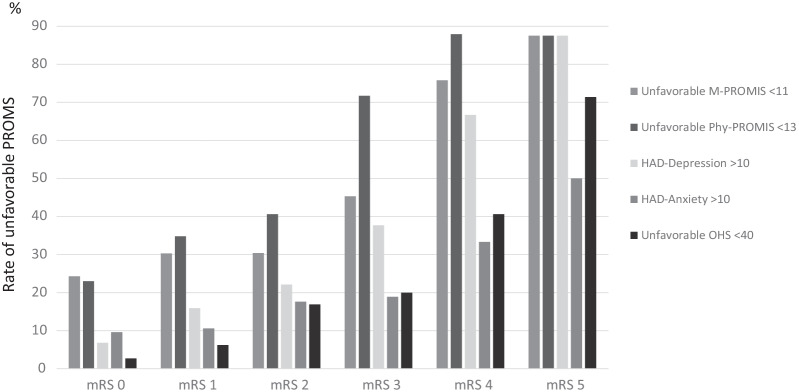
Rate of unfavorable results in the different PROMs evaluated at 3 months after stroke related to the 3 months mRS score

All PROMs evaluated at 3 months showed a mild to moderate correlation with mRS measured at the 3 months follow-up out-patient visit: HADS-depression, rho = 0.39; HADS-anxiety, rho = 0.26; PHY-PROMIS, rho = 0.40; M-PROMIS, rho = 0.28 and OHS rho = 0.48 (p < 0.01 for all correlations). However, even patients with a favorable mRS (< 3) presented a relevant rate of unfavorable results in the measured PROMs: 8% of OHS < 40, 15% HAD-depression, 12.1% HAD-anxiety, 28.7% poor mental results in M-PROMIS and 33.1% unfavorable PHY-PROMIS. Plausibly, patients with worse mRS showed extremely poor results in PROMs: only 14% of patients with a mRS > 2 reported all favorable results in PROMs, as compared with 55.2% of patients with mRS < 3. Figure 7 depicts the progressive increase in the rate of unfavorable results in PROMs according to the growing mRS scores at 3 months.

We performed 2 multivariate regression models to identify independent predictors of unfavorable results in PROMs at 3 months, shown at Table 2. The first model included variables determined during hospitalization and at discharge, adjusted by known baseline predictors of functional outcome (age, sex, stroke subtype, baseline and discharge NIHSS, mRS at discharge) and also by discharge destination (to home or to a socio-rehabilitation center (SRC)). To note, female sex and a higher mRS at discharge predicted all unfavorable results in PROMs (p < 0.01) at 3 months. Discharge to a SRC instead of home also predicted poor mental self-perception measured by M-PROMIS and depression at 3 months (p < 0.01 for both), and stroke severity measured by the NIHSS at discharge predicted unfavorable OHS (p = 0.018).

**Table 2:** Significant 3 months-PROMs predictors in two multivariate regression models

	Discharge predictors			Discharge + 7 days PROMs predictors		
	OR	95% CI	p	OR	95% CI	P
*Poor results 90 days PHY-PROMIS (< 13)*						
Sex (male)	0.42	0.28–0.62	< 0.001	0.44	0–27-0.71	0.001
Discharge mRS	1.80	1.55–2.10	< 0.001	1.31	1.07–1.59	0.007
M-PROMIS 7 days	n.a			2.42	1.42–4.12	0.001
PHY-PROMIS 7 days	n.a			5.27	3.11–8.94	< 0.001
*Poor results 90 days M-PROMIS (< 11)*						
Sex (male)	0.50	0.34–0.73	< 0.001	0.57	0.36–0.93	0.023
Discharge NIHSS	n.s			1.11	1.03–1.19	0.005
Discharge mRS	1.43	1.20–1.71	< 0.001	n.s		
Discharge SRC	1.97	1.08–3.58	0.026	n.s		
OHS 7 days	n.a			0.98	0.97–0.99	0.001
M-PROMIS 7 days	n.a			5.27	3.15–8.80	< 0.001
*HAD-depression (≥ 10)*						
Sex (male)	0.50	0.34–0.73	< 0.001	0.55	0.32–0.95	0.033
Discharge mRS	1.53	1.25–1.86	< 0.001	1.37	1.08–1.72	0.008
Discharge SRC	2.44	1.60–5.59	0.005	n.s		
M-PROMIS 7 days	n.a			4.85	2.66–8.86	< 0.001
PHY-PROMIS 7 days	n.a			2.52	1.23–5.17	0.011
OHS 7 days	n.a			0.98	0.97–0.99	0.006
*HAD-anxiety (≥ 10)*						
Sex (male)	0.43	0.26–0.69	0.001	0.44	0.25–0.75	0.003
Discharge mRS	1.48	1.26–1.74	< 0.001	n.s		
M-PROMIS 7 days	n.a			5.74	3.24–10.14	< 0.001
*Poor results OHS (< 40)*						
Age	1.02	1.00–1.05	0.037	n.s		
Discharge NIHSS	1.10	1.02–1.20	0.018	1.15	1.07–1.24	< 0.001
Discharge mRS	1.46	1.07–1.99	0.017	n.s		
M-PROMIS 7 days	n.a			3.29	1.60–6.76	0.001
OHS 7 days	n.a			0.96	0.94–0.97	< 0.001

The first model includes variables at discharge, and the second one adds PROMS acquired after 7 days from discharge

*PROMs* patient reported outcomes; *OR* odd ratio; *CI* confidence interval; *PHY-PROMIS* physical-PROMIS; *mRS* modified Rankin Scale; *NIHSS* National Institute of Health Stroke Scale; *SRC* socio-rehabilitation center; *M-PROMIS* mental-PROMIS; *OHS* overall health status; *n.a.* not applicable; *n.s.* not significant

The second multivariate regression model added to the previous variables the results of PROMs acquired 7 days after discharge. As expected, all early unfavorable results in PROMs measured at 7 days were strong independent predictors of the corresponding 3 months PROMs (p < 0.01 for all). In the case of HADs, this scale was not acquired at 7 days in accordance with ICHOM. M-PROMIS score emerged as a strong predictor of both depression and anxiety determined by HADS at 3 months (p < 0.001 for both).

## Discussion

We have established a PROMs collection program for stroke patients discharged from our Comprehensive Stroke Center and have detected a high proportion of stroke survivors who report poor outcomes 3 months after the event, even in patients with favorable mRS and frequently in several outcome domains. We were able to identify female sex, discharge to a SRC and especially poor results in PROMs evaluated after 7 days as independent predictors of poor outcomes reported by patient 3 months after the stroke.

Classically, outcome determination after an acute ischemic stroke has been performed by the modified Rankin scale evaluation, measured by a certified stroke expert (neurologists) usually 3 months after index event. It is considered an extremely useful tool to evaluate results in clinical trials and allows easy comparison between treatment strategies. However, it is probably too coarse and mainly focused on motor disability and, therefore, it does not entirely reflect the most valuable health determinants for patients. In fact, our results suggest that even patients with favorable mRS present poor results in the PROMs surveys, remarking the need for more sensitive outcome measures. Our PROMs selection fulfills ICHOM Standards, allowing benchmarking, but it was also defined by interviews with stroke patients and families, to better reflect what they considered important for their well-being. Our PROMs recollection program is framed within a value-based stroke care management, and in the selection process was also considered surveys aimed to detect the main problems of the patients with the objective to identify them early and design new care pathways to improve their health perception. In addition to surveys recommended by ICHOM, specific PROMs related to patient’s characteristics (age, gender, work status, etc) could be considered, and at the moment we are establishing a more individualized PROM recollection.

PROMIS-10 is a scale specifically developed to evaluate the global self-perception of health by patients, including pain, fatigue, and social health. Almost half of our stroke patients have a poor perception about their physical health status measured through the PHY-PROMIS after 3 months, and the majority remain with the same impression after one year. Similarly, in the case of M-PROMIS, 39.3% of patients presented poor outcomes at 3 months and 33.3% after a year. The overall health status (OHS) shows a statistically significant, but only mild, improvement along the year of follow-up. This statistical improvement may not even reflect a clinically relevant improvement of the patient’s self-perception of health. These results show that a great percentage of the patients consider their health status poor 3 months after the stroke, but also that there may be lack of clinically relevant improvement for the patients later-on. Furthermore, the logistic regression analysis including PROMs at day 7 after discharge show that these early results on PROMs are the strongest predictors of PROMs at 3 months as compared with other relevant variables. Therefore, new treatment strategies are required, based on the patient’s needs, that could be designed before discharge or early afterwards according to the 7 days PROMs. Previous studies have shown the effectiveness of conducting an interview with the patient/family during hospital admission to assess the environment in which they live and where they normally carry out their occupations. After being discharged home, an individualized physical rehabilitation treatment directed to the needs that patients’ activities require may result in better outcomes [13, 14]. PROMs evaluation may help to develop these new treatment strategies by the identification of the most relevant problems for patients, but they should also be considered as efficacy outcomes when designing new clinical trials.

Stroke is a disease that requires comprehensive and integrated care and an interdisciplinary approach, given its complexity [15]. Programs focused on ongoing comprehensive care from the onset of hospitalization to the patient’s home have been shown to accelerate discharge and increase the likelihood of long-term independence. The effectiveness of these programs depends on the correct selection of patients and coordination with community and social services [16]. The average life expectancy for the general population is constantly increasing [17]. In 2017, 18.63% of the population was 65 years or older; the forecast is growing with a projection of 30.8% in 2050 [18] and the legitimate aspiration to enjoy a better quality of life highlights the need to develop innovative lines of research aimed at promoting mobility and functional independence of people who have suffered a stroke and assess psychosocial wellbeing, with the secondary objective of reducing the social and economic burden that disability entails.

Our study also detected a high proportion of patients with depression and anxiety after stroke. A limitation of the study is that we did not consider the diagnosis of depression or anxiety before the stroke occurrence. However, post-stroke depression is one of the aftermaths best known and studied worldwide. A recent meta-analysis estimates that around fifteen million people that suffer a stroke per year could develop depression [19]. However, depression is not actively sought by most physicians taking care of stroke patients, and therefore, these patients are being underdiagnosed or not correctly treated. Furthermore, after one year, from all patients with pathological punctuation on the HADS scale at 3 months, almost half continued reporting depression and anxiety. PROMs collection programs including scales aimed to detect these psychiatric complications may improve their detection and management, and therefore impact on the patients’ functional outcomes [20, 21].

The study also shows several predictors of unfavorable PROMs that can be already identified during hospitalization and at discharge. As expected, mRS at discharge predicted all PROMs after 3 months. One of the most relevant results is that destination to social-rehabilitation center (SRC) instead of home predicted poor M-PROMIS and depression. Numerous studies demonstrate the importance of the home environment in the rehabilitation of stroke patients. Not only does it facilitate functional recovery, but it also helps the patient to perceive in a more positive way their recovery process and state of health [22].

On the other hand, it should be noted that female sex emerged also as an independent predictor of poor result in PROMs. Globally, anxiety and depression present higher prevalence in women, which may explain part of our results. However, several studies have also shown that there is a greater morbidity and mortality from stroke in women [23, 24] In addition to pure biological causes, the historical role of women as caregivers may generate a greater biopsychosocial impact [25]. Whether specific sex therapeutic approaches and further social covert in women may influence these results warrant discussion.

The main limitation of our study was the decrease in PROMs compliance during the follow up. Seven days after hospital discharge, 32% of patients did not complete the PROMs questionnaires. The lack of fulfillment increased to almost half at 3 months and 75% after one year from stroke. We hypothesized that this lack of compliance may be partly caused because patients consider that no therapeutic action is taken with their results. A multidisciplinary approach with real time interaction between the patient and different health providers (neurologist, physical and occupational therapist, psychologist and primary care physicians) may improve this perception. Patients and health care providers should act as a team to decide the best individual treatment for each person according to different data, including PROMs. Given these results, the importance of reaching the patients more effectively and making them an active participant throughout the process was considered. Therefore, a new recollection tool based on a mobile application that was designed in our center for treatment compliance and health education has been developed [26]. NORA is a web platform with an app for patients that includes several functionalities such as tele-rehabilitation modules and a chat that allows real-time two-way communication. We believe that improving communication and patient’s implication on their own health will also improve the PROMs compliance and are at the moment performing a clinical study to validate it.

## Conclusion

In conclusion, our PROMs collection program, based on ICHOM standards of survey recollection at scheduled time-points along out-patient follow-up, detected a high rate of poor physical and mental self-reported outcomes as well as depression and anxiety 3 months after the stroke, even in patients with favorable mRS. There was not a significant improvement in most PROMs results during follow-up. PROMs fulfillment decreased over time, showing a need for new strategies to increase patient attachment. Female sex, mRS and discharge to SRC predicted the results of PROMs at discharge, but the most potent predictors of 3 months PROMs results were the results of PROMs recollected at day 7.

## Data Availability

The data that support the findings of this study are available from the corresponding author on reasonable request.
